# Findings in Chinese Patients With Parkinson's Disease: A Content Analysis From the SML Study

**DOI:** 10.3389/fpsyt.2021.615743

**Published:** 2021-02-02

**Authors:** Yiwei Qian, Yi Zhang, Xiaoqin He, Shaoqing Xu, Xiaodong Yang, Chengjun Mo, Xiaomeng Lu, Mengjuan Qiu, Qin Xiao

**Affiliations:** ^1^Department of Neurology, Ruijin Hospital, Shanghai Jiao Tong University School of Medicine, Shanghai, China; ^2^Department of Digital, Huimei Digital Tech (Beijing) Co., Ltd, Beijing, China

**Keywords:** Parkinson's disease, social media, burdens, management, care-giver

## Abstract

Social media listening (SML) is a new process for obtaining information from social media platforms to generate insights into users' experiences and has been used to analyze discussions about a multitude of diseases. To understand Parkinson's disease patients' unmet needs and optimize communication between doctors and patients, social media listening was performed to investigate concerns in Chinese patients. A comprehensive search of publicly available social media platforms with Chinese-language content posted between January 2005 and April 2019 in mainland China was performed using defined Parkinson's disease-related terms. After multiple steps of machine screening were performed, a series of posts were derived. The content was summarized and classified manually to analyze and map psychological insights, and descriptive statistics were applied to aggregate findings. A total of 101,899 patient-related posts formed the basis of this study. The topics mainly focused on motor symptoms (*n* = 54,983), choice of pharmaceutical drugs (*n* = 45,203) and non-motor symptoms (*n* = 44,855). The most common symptoms mentioned were tremor (54.5%), pain (22.9%), and rigidity (22.1%). Psychological burden (51%) and work/social burden (48%) were the most concerning burdens for patients and their families. The compound levodopa (43%) and dopamine agonists (23%) were the most common options for the patients, while concerns about new-generation anti-Parkinson's disease medication increased. The portraits of patients suggested varying characteristics across different periods and advocate for personalized service from doctors. In the management of patients, it is imperative to plan individualized therapy and education strategies as well as strategies for social support.

## Introduction

Parkinson's disease (PD) is the second most common neurodegenerative disease, ranking lower only than Alzheimer Parkinsons disease (1), and affects more than 10 million people worldwide. The average prevalence of PD in the Han population in China is ~3.9‰ of those ≥50 years of age (2). It is estimated that the number of Chinese patients with PD will increase to 4.94 million by 2030, accounting for half of PD patients worldwide (3). This disease has a relentless and progressive course that results in motor symptoms, including resting tremor, bradykinesia, limb rigidity, and gait and balance problems, and a series of non-motor manifestations, such as loss of olfaction, psychiatric symptoms, autonomic dysfunction (e.g., sexual dysfunction, gastrointestinal disorders, orthostatic hypotension), sleep disorders, mild cognitive impairment and dementia (4). The symptom burden in patients with PD imposes a significant challenge that has a substantial impact on patients' physical and psychological well-being (5).

The Internet has transformed communication channels for people in general and patients in particular, with social media interactions including online forums, microblogs, social networks and content communities (6). A growing body of literature focuses on investigating the use of social media, defined as a group of online applications that allow for the exchange of unverified user-generated information among patients and caregivers, as a source of health information (7). Social media platforms provide a window into patients' perceptions of their diseases, their satisfaction with outcomes, and other factors that affect their lives (8). Social media listening (SML) is a new approach to harnessing information derived from social media platforms to generate insights into users' experiences (9). It has been used to investigate discussions on diverse diseases, including multiple sclerosis (10), dry eye disease (8), inflammatory bowel disease (11), and chronic obstructive pulmonary disease (6). Previous studies have evaluated and assessed the influence of Chinese video-based lectures about PD on YouTube™ (12). YouTube™, Facebook™, and Twitter™ are popular around the world but are not available in China. With the rapid development of the Internet and mobile apps in China, web-based consultations could serve as an effective communication tool in conventional physician-patient relationships and have already acted as a supplementary tool for traditional health care since 1999 (13).

To date, there is limited literature on the use of SML to investigate the needs and experiences of Chinese patients with PD. The aim of the current study was to perform a content analysis study based on SML to provide insights into disease burden, diagnosis, treatment patterns and quality of life in Chinese patients with PD.

## Materials and Methods

### Study Design and Data Sources

**Figure 1:**
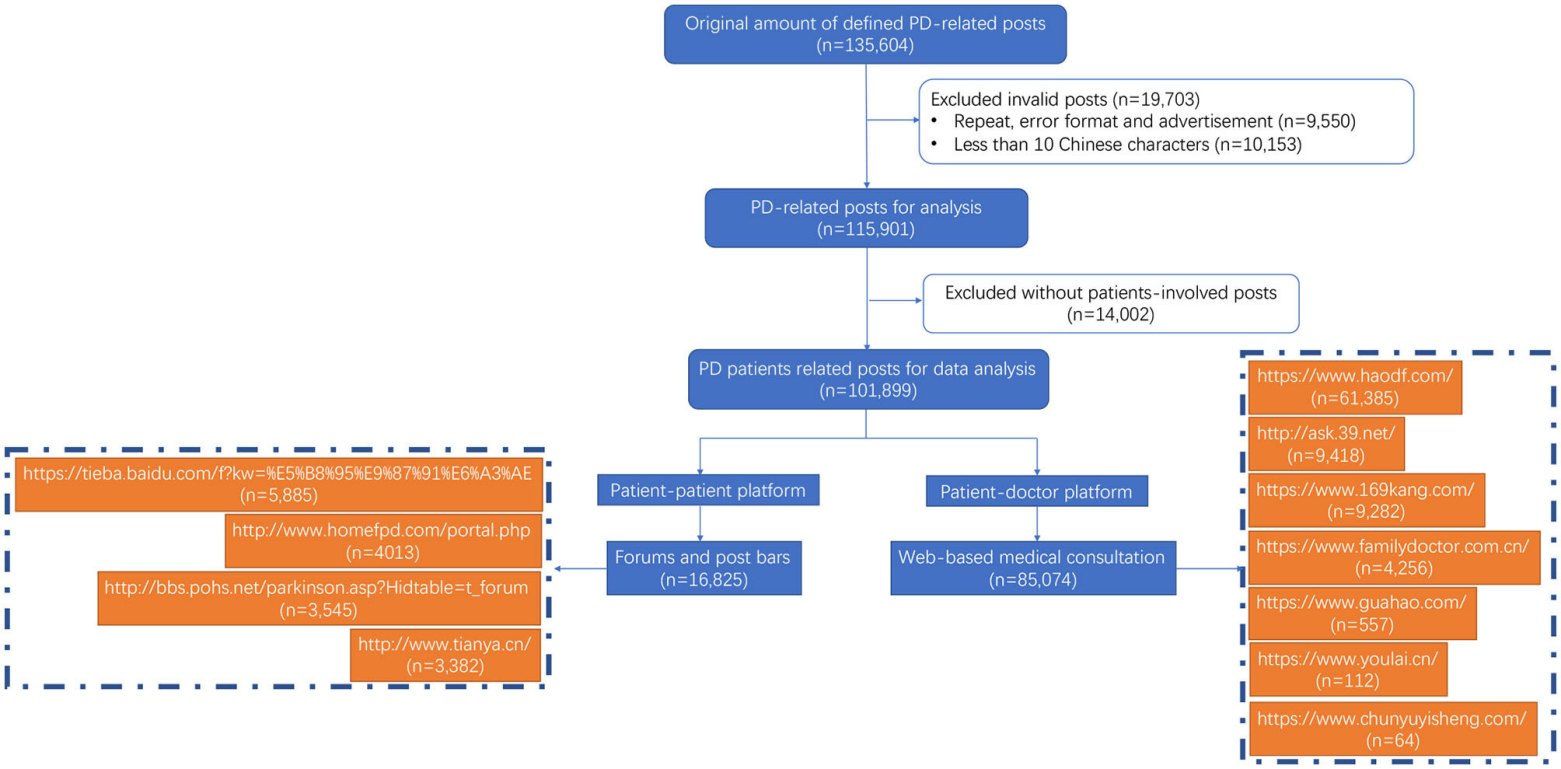
Flow chart of post selection and tagging and domain names of the websites from which posts were extracted.

A comprehensive search was performed on social media platforms, including patient-patient discussion platforms (mainly forums and post bars) and patient-doctor inquiry platforms (mainly web-based medical consultations) (Figure 1). Online posts in the Chinese language that appeared between January 2005 and April 2019 in mainland China were collected and evaluated using the following predefined search terms: “PD” or “Parkinson” or “Parkinsonism.” Social media platforms such as Weibo (like Twitter in China) were excluded because of a lack of public access.

### Ethical Considerations

All data utilized and presented in this study were obtained from publicly accessible sources without accessing password-protected information. The privacy of patients was respected, and caution was taken in using the information posted by them; all online content was anonymized in compliance with data privacy obligations. We declare that no individual patient data requiring consent have been presented. This study was reviewed by the Research Ethics Committee, Ruijin Hospital affiliated with Shanghai Jiaotong University School of Medicine, and it was deemed exempt from review as it did not meet the definition of “human or animal subject research.”

### Filtering of Posts and Text Data Cleaning

The desired information was collected from the two predefined online data sources using a pair of Web crawlers designed to extract a set of predefined key words related to symptoms, diagnosis, medication, treatment, complications, and management. Social Studio® is an online aggregator tool that provides downloadable links of posts from social media channels based on specific keywords. Links available from Social Studio® were accessed, and the posted content was retrieved in Microsoft Excel (Microsoft, Redmond, WA, USA) and then anonymized and cleaned manually to filter out duplicates. The term “post” was defined as social media content with a unique identifier. “Record” was defined as a unique individual (patient or caregiver) who was identifiable by the same content even when his/her posts had different time stamps. “Mention” was defined as the number of times a parameter was mentioned, which means the total number of times a symptom, treatment or other parameter was mentioned irrespective of the number of posts. The content of each post was manually anonymized and cleaned to filter out duplicates and advertisements. Posts with formatting errors or fewer than 10 Chinese characters in the title combined with the following criteria were also excluded as invalid information. Of the 135,604 retrieved posts, 19,703 were excluded based on the selection strategy (Figure 1).

### Categorization and Indexing of Social Media Posts

Information was extracted using natural language processing (NLP) techniques, traditional keyword analysis, and computer selection. Traditional keyword analysis was performed to further extract the content, followed by keyword generalization to cover more records. Demographic information about users, including sex, age, and disease course, was recorded or inferred from the content when possible. Descriptions about patients' perceptions of aspects associated with PD were summarized and mainly included but were not limited to symptoms, diagnosis, management and impact on patient quality of life. All manual data cleaning was performed following a four-step review (self-review, peer analyst review, senior analyst review and team review) to ensure the robustness of the analysis and tagging. Posts were further analyzed to determine the “number of mentions” of a particular theme in the posts.

### Attention Score

The attention score of each theme was computed using the following equation and the fold ratio of the posts:

(1) $$\begin{array}{l} Attention_{ij}=\frac{A_{ij}}{\sum_{m=1}^{M}A_{mj}\;\cdot \;\sum_{n=1}^{N}A_{in}}\cdot \sum_{m=1}^{M}\sum_{n=1}^{N}A_{mn} \end{array}$$

The attention score reflected the deviation of the actual value from the maximum likelihood estimate and indicated the difference between the true value and the expected value as follows: 1 indicated that the true value equaled the expected value and that the degree of attention was moderate, >1 indicated that the true value was greater than the expected value and the degree of attention was high, and <1 indicated that the true value was less than the expected value and the degree of attention was low.

### Data Analysis

All data were analyzed using descriptive statistics. Categorical data are described using the number of posts and/or percentages. For the anonymized content, only aggregated qualitative findings are reported. Manual curation was used to analyze and map the psychological aspects expressed by users.

## Results

### General Data Analysis

**Figure 2:**
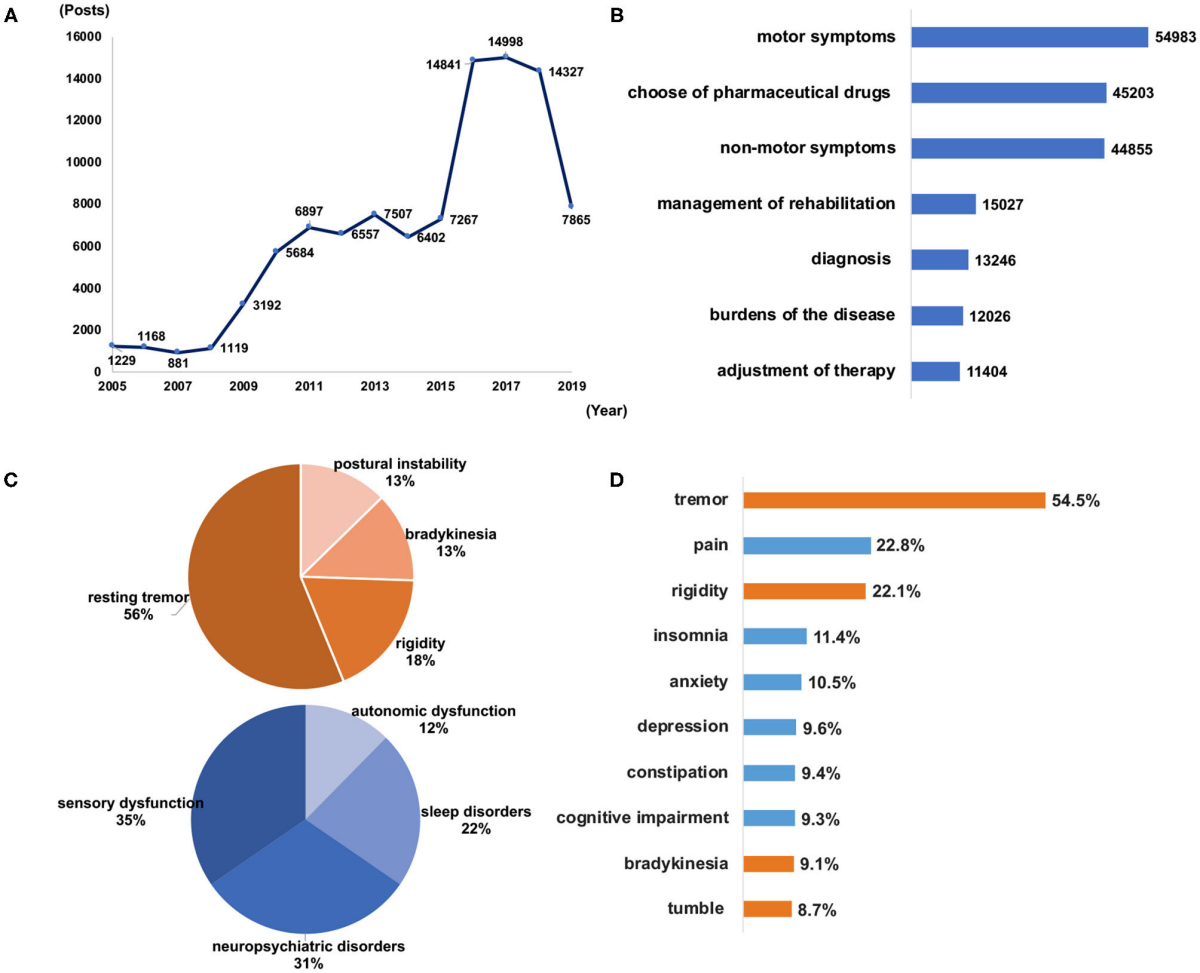
The number of included posts made from 2005 to 2019 **(A)** and the major topics of discussion on social media according to the number of mentions in the posts **(B)**. Mentions in posts pertaining to the classification of motor symptoms and non-motor symptoms **(C)** and the top 10 symptoms mentioned by the patients **(D)**. Orange bars indicate motor symptoms, and blue bars indicate non-motor symptoms.

Among the 101,899 identified posts, the patient-doctor platform was the primary source of information and contributed 83% of the total posts, followed by the patient-patient platform (Figure 1). Most of the social media posts were produced in Beijing (36%) and Shanghai (13%), followed by Jiangsu Province, Henan Province and Guangdong Province (30,878 posts, Supplementary Figure 1). It is worth noting that the number of online posts increased significantly from 2015 to 2016 (Figure 2A). Sex could be inferred in 35,889 records, and there were similar numbers of male (46%) and female (54%) patients. Age was available from 21,617 records; 78% of patients were between 50 and 80 years old, while 31% of patients were between 60 and 70 years old. Eighteen percent of the patients were aged ≤50 years, and these patients had a higher mention rate of keywords such as “family history,” “genetic,” and “young onset.” The disease duration since diagnosis was available for 8,937 unique records, with 46% having a duration of fewer than 5 years, 39% having a duration of 5–10 years, and 15% having a duration of more than 10 years.

### Extracted Major Topics

The major themes in the posts were “motor symptoms,” “choice of pharmaceutical drugs,” “non-motor symptoms,” “management of rehabilitation,” “diagnosis,” “burden of the disease,” and “adjustment of therapy” (Figure 2B). Patients whose disease duration was fewer than 5 years were concerned about “diagnosis,” “management of rehabilitation” and “motor symptoms;” patients whose disease duration was 5–10 years paid more attention to “adjustment of therapy,” “choice of pharmaceutical drugs,” “non-motor symptoms,” and “burden of the disease;” and patients whose disease duration was longer than 10 years were concerned about “burden of the disease,” “adjustment of therapy,” “non-motor symptoms” and “choice of pharmaceutical drugs” (Table 1). The different distributions on the two platforms are shown in Supplementary Figure 2.

**Table 1:** Attention to topics involved by disease duration.

**Topic**	**N**	**0–5 years**	**5–10 years**	**>10 years**
**Major themes**				
Motor symptoms	5,708	1.032	0.971	0.978
Non-motor symptoms	5,440	0.986	1.003	1.035
Choice of pharmaceutical drugs	4,894	0.990	1.011	1.002
Adjustment of therapy	2,407	0.881	1.108	1.082
Burden of the disease	1,666	0.912	1.002	1.209
Management of rehabilitation	1,398	1.150	0.926	0.738
Diagnosis	946	1.159	0.919	0.729
**Motor symptoms**				
Resting tremor	4,854	0.999	1.015	0.965
Postural instability	1,257	0.896	1.025	1.267
Rigidity	954	0.982	1.037	0.969
Bradykinesia	889	1.168	0.842	0.85
**Non-motor symptoms**				
Neuropsychiatric disorders	3,257	0.967	1.022	1.035
Sensory dysfunction	2,853	1.048	0.957	0.973
Autonomic dysfunction	2,089	1.02	0.978	0.999
sleep disorders	1,765	0.959	1.054	0.981
**Burden of the disease**				
Work burden	692	0.854	1.097	1.135
Psychological burden	472	1.158	0.945	0.743
Social burden	189	1.137	0.782	1.149
**Potential motor complications after levodopa treatment**				
Dyskinesia	5,832	1.141	1.005	0.415
On-off phenomenon	501	0.668	1.133	1.447
wearing-off phenomenon	322	0.772	1.12	1.239
**Potentially unresolved symptoms after levodopa treatment**				
Pain	2,047	1.037	0.983	0.949
Rigidity	1,882	1.055	0.969	0.936
Depression/anxiety	1,300	0.971	1.018	1.031
Constipation	734	0.98	0.999	1.053
Sleep disorders	708	0.919	1.063	1.05
Falls	675	0.898	1.039	1.162
**Potentially unresolved symptoms after dopamine agonists treatment**				
Pain	1,253	1.043	0.993	0.908
Rigidity	1,107	1.071	0.961	0.921
Depression/anxiety	814	0.968	1.001	1.078
Constipation	427	0.954	1.040	1.013
Sleep disorders	454	0.919	1.066	1.035
Falls	445	0.888	1.008	1.264

*The degree of attention was calculated as the fold ratio of the posts for each theme*.

= *1, the true value is equal to the average expected value, and the degree of attention is moderate*;

>*1, the true value is greater than the average expected value, and the degree of attention is high; <1, the true value is less than the average expected value, and the degree of attention is low*.

### Perception of Motor and Non-Motor Symptoms

Among the 54,983 posts related to motor symptoms, the reported symptoms were “resting tremor,” “rigidity,” “bradykinesia” and “postural instability.” In the 44,855 posts related to non-motor symptoms, the reported symptoms were “sensory dysfunction” (including pain, numbness, and disturbance of sensation), “neuropsychiatric disorders” (including anxiety, depression, mania, hallucination, apathy, and irritability), “sleep disorders” (including insomnia, daytime sleepiness with sleep attacks, restless legs syndrome and REM sleep behavior disorder) and “autonomic dysfunction” (including sexual dysfunction, swallowing and gastrointestinal disorders, bowel and bladder abnormalities, and derangements of cardiovascular regulation) (Figure 2C). Of the top 10 most frequently mentioned symptoms, the top 3 were tremor, pain and rigidity (72,346 posts, Figure 2D), which were also the most common symptoms across different age groups (Supplementary Figure 3A). In the 36,376 posts with known sex, all symptoms were mentioned more in female than in male patients, especially “pain,” “insomnia,” “depression,” and “anxiety” (Supplementary Figure 3B). The emotions expressed referring to symptoms were also analyzed. Negative emotions, including “nervous,” “unhappy,” and “worried,” were more commonly used in reference to non-motor symptoms than to motor symptoms (97 vs. 68%). The emotions expressed were associated more with the symptoms and disease, based on the causal relationship, than with the “depression” symptom itself. As the disease progressed, the proportion of positive emotions showed an upward trend, and the negative emotions showed a downward trend. However, negative emotions were always mentioned more often than positive emotions by patients (Supplementary Figure 4).

### Perspectives on Burden

Patients' perceptions of burden were assigned to four major categories: psychological burden, work/social burden, caregiver burden and economic burden (12,170 posts, Supplementary Figure 5A). In terms of the psychological burden, “anxiety” and “depression,” both of which are viewed as negative emotions, had the highest mention rates (6,198 posts, Supplementary Figure 5B). “Cognitive impairment” (including memory loss, get loss, and dementia) and “language impairment” (including speech difficulties and disfluency in talking) were the main causes of social burden (4,704 posts, Supplementary Figure 5C). Female patients had a higher psychological and social burden than male patients, while male patients suffered more from workload burden than female patients (5,541 post, Supplementary Figure 5D). Patients whose disease duration was fewer than 5 years were the most concerned about “psychological and social burden.” Patients whose disease duration was over 10 years were concerned most about “work/social burden” (Table 1).

### Diagnosis and Treatment

In terms of assisting in clinical diagnosis (11,404 posts), 82% of auxiliary examinations focused on neuroimaging examinations, and 18% mentioned laboratory tests. A small percentage of patients mentioned the acute L-dopa challenge test (LDCT) (118 posts), which involves rating motor symptoms according to the Unified Parkinson's Disease Rating Scale (UPDRS III) score both before and after a “single shot” administration of an above-threshold dose of L-dopa, enabling this criterion to be tested in a standardized way (14). Drug treatment was the major treatment option for managing PD (45,203 posts), followed by surgical therapy (12,502 posts) and non-surgical or drug therapy (6,509 posts). For pharmaceutical drugs (Supplementary Figure 6A), the most commonly mentioned were levodopa (44%; Madopar® for 78% and Sinemet® CR for 22%) and dopamine agonists (22%). Anticholinergic drugs had a 13% mention rate, while amantadine had an 11% mention rate. In terms of monoamine oxidase B (MAO-B) inhibitors (5%), selegiline accounted for 81%, while the second-generation drug rasagiline accounted for 34%. With regard to COMT inhibitors (5%), entacapone was mentioned in 94% of posts, while entacapone, levodopa and carbidopa tablets accounted for only 6%. With disease progression, the posts related to levodopa showed a slight downward trend, and dopamine agonists and COMT inhibitors showed a tendency to increase (Supplementary Figure 6B). In terms of initial medication, the patients were more concerned about “levodopa.” Although attention to this treatment as a modified medication has declined, “levodopa” remains the main treatment for patients; while, the records related to dopamine agonists and COMT inhibitors tended to increase (5,203 posts, Table 2). Patients with a disease duration of fewer than 5 years were more concerned about “dyskinesia.” Patients whose disease duration was over 10 years were more concerned about the “on-off phenomenon” and “wearing-off phenomenon” than about “dyskinesia” (Table 1). Regardless of whether treatment involved levodopa or dopamine agonists, “pain,” “rigidity,” and “depression” were also the most common unresolved symptoms in the included patients (Table 1).

**Table 2:** Attention scores for topics by medication adjustment.

**Topics**	**Initial medication (*N* = 1,910)**	**Modified medication (*N* = 5,203)**
Levodopa	1.265	0.903
Dopamine agonists	0.384	0.634
Anticholinergic drugs	0.338	0.245
Amantadine	0.303	0.302
MAO-B inhibitors	0.134	0.140
COMT inhibitors	0.116	0.235

For other management strategies, patients with a disease duration of fewer than 5 years were more concerned about “self-rehabilitation management” (including different kinds of rehabilitation training, self-exercise, and traditional Chinese medicine treatments, such as Ding Zhen Pill—currently known as Ding Zhen Decoction—Chinese dietary therapy, acupuncture, moxibustion, Tai Chi, and Qigong) (18%, 4,145 posts), and attention to this topic gradually decreased as the disease progressed, with patients whose disease duration was over 10 years having a mention rate of only 12% (12%, 1,305 posts) (Supplementary Figure 6C). Patients mainly preferred traditional Chinese medicine treatment, physical therapy, and low-strength functional training, e.g., Tai Chi, walking, and jogging.

### Quality of Life

Social barriers had the greatest impact on quality of life, and they included “stigma,” “inability to engage in social activities” and “loneliness/apathy” (6,168 posts). Overall, 2,805 posts were related to concerns about daily activity and covered ~9 problems, among which “incontinence,” “inability to walk,” and “inability to eat” were the most common (Supplementary Figure 6D).

## Discussion

**Figure 3:**
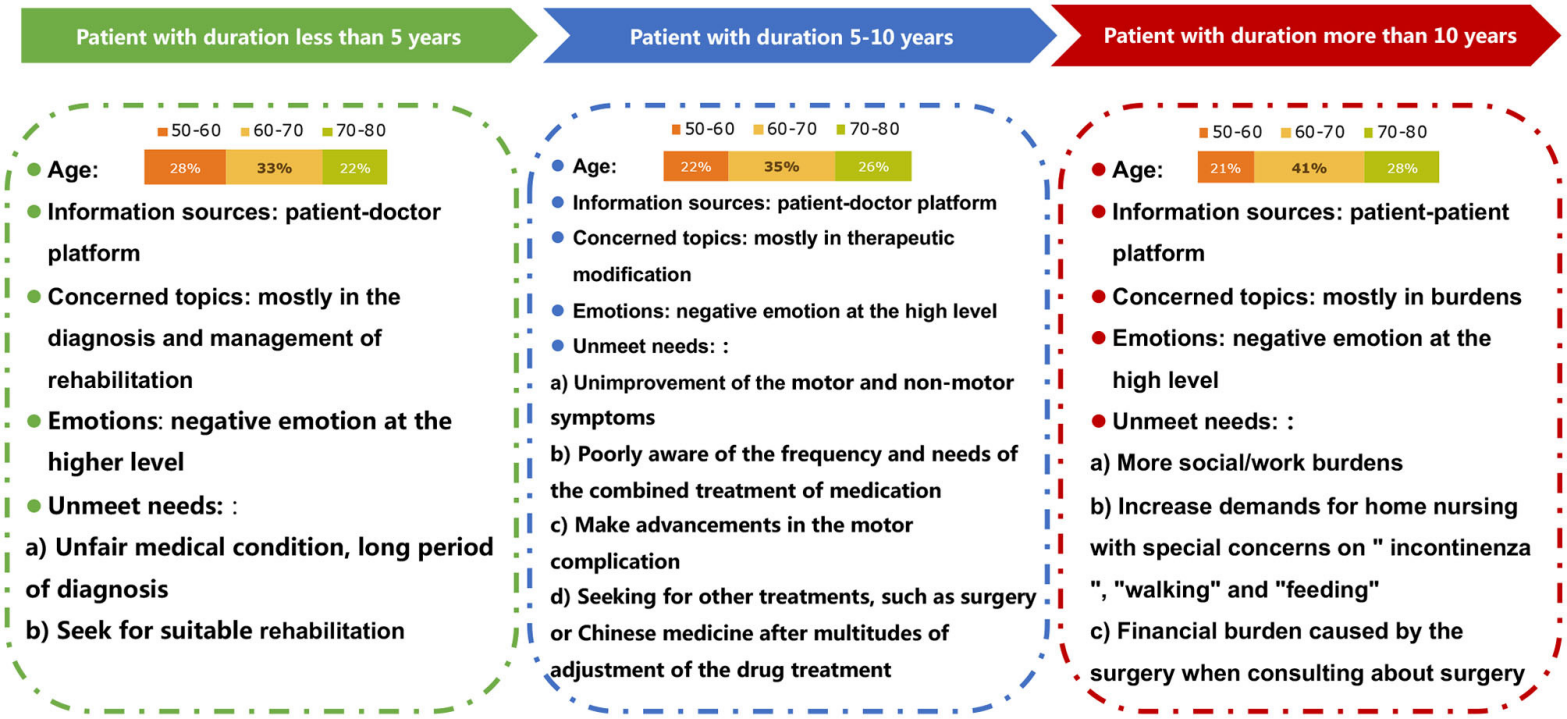
Portraits of PD patients showing that those with different disease durations presented different characteristics, suggesting the need for personalized patient care and management.

Our research represents the first study on the usefulness of social networks in exploring issues related to Chinese patients with PD. Based on the above investigations, portraits of PD patients can be drawn that illustrate that patients with different disease durations present different characteristics, suggesting the need for doctors to provide personalized patient care and management (Figure 3). In the early stage, patients want to know more about whether they have a confirmed diagnosis of PD, whether their motor symptoms meet the diagnostic criteria of PD or not, and how suitable rehabilitation can be obtained early in the disease course, and they exhibit more negative motion. As their disease progresses, patients are more concerned about the modification of therapy, given the emergence of motor complications and non-motor symptoms, and after several adjustments of the drug treatment, they seek other treatments, such as surgery or traditional Chinese medicine. Advanced PD patients experience the heaviest social/work burdens. Their need for home nursing increases, and they express special concerns about “incontinence,” “walking,” and “feeding” and the financial burden caused by the surgery.

The online PD patients were mainly over 60 years old, consistent with epidemiological data from China (15). As the economic centers of China, Beijing and Shanghai have a large number of top hospitals and numerous famous doctors who are trusted by most patients. Beijing and Shanghai also have higher online web-based consultation rates than have been found in other provinces (13). Given the limited medical resources in developing regions, it is necessary to raise patients' sense of self-management and increase the use of online consultations for more PD-related online activities. The online posts show that patients thought that the process of being diagnosed with PD was difficult and time-consuming, consistent with the fact that PD is commonly missed or misdiagnosed, as there is no available specific diagnostic test (16).

Postural instability or balancing issues are the primary motor symptoms that emerge in the late stages of PD (17), consistent with our results. Motor symptoms were mentioned slightly more often than non-motor symptoms. As the disease progressed, the proportion of positive emotions showed an upward trend, suggesting that with knowledge of the disease and the positive effects of medication, patients build confidence and become satisfied with the treatment, especially in the “honeymoon” phase. However, the patients always exhibited more negative than positive emotions, particularly in the earlier stages. Therefore, emotional management of patients should be carried out as soon as possible to improve the overall treatment experience and quality of life of patients. Additionally, negative emotions were more frequently generated as a result of non-motor symptoms. This indicates that physicians need to pay more attention to the occurrence of non-motor symptoms, especially pain, which was the sensory impairment most commonly reported by patients, exceeding mentions of constipation, mood disorder or unexpected olfactory impairment. A previous study showed that pain was ranked highly as a troublesome symptom in all stages of the disease and was particularly frequent in early-stage PD (18, 19). Separating PD-related pain from pain of other origins is an important challenge due to a phenomenon known as “many syndromes under the same umbrella” (18); ~40% of patients may not report this complaint in routine visits to a physician (20). Most epidemiological data are based on questionnaires that were not specifically validated for PD patients. The different focuses of patients and doctors strongly attracted our notice. Additionally, sex-related differences have been reported in clinical phenotypes (21), consistent with our study. Female sex has been confirmed to be associated with more common, severe, persistent or episodic pain (22, 23) and anxiety/depression (24, 25) in previous clinical studies. Sleep disorders are also common in PD, and inconsistent with the results of clinical studies, the prevalence has been reported to be higher in men than in women (26, 27). Based on the sex-related effects of PD on non-motor symptoms, female PD patients should pay more attention to negative emotions, for which they may need guidance from clinical staff and caregivers (28).

One of the key themes was an expression of the need for better medications due to patients' frustration with the relief offered by currently available medications. Patients exhibit substantial medication non-adherence. To extend the “honeymoon period,” self-adjustment and the discontinuation of levodopa are prevalent in patients and might cause accelerated progression or result in motor complications (29). Motor complications take various forms in different stages, including wearing off during the early stage, dyskinesias in the intermediate stage, and complex fluctuations in the advanced stage (30). Interestingly, patients in the early stage fear dyskinesias, which are described as an experience of involuntary movements by some patients or in online news. In the advanced stage, patients' feelings of dissatisfaction increased with the severity of OFF time. As the disease progresses, many patients may exhibit poor self-awareness about experiencing mild dyskinesia that may not necessarily need to be treated, and patients often do not notice the presence of dyskinesia unless it becomes disabling (31). Physicians are required to not only choose appropriate PD therapeutic drugs and prescribe the correct dosages but also prescribe PD medication in a way that considers patient's feelings (32). Therefore, targeted interventions, such as scientific guidance and follow-up management, should be developed and strengthened in PD patients.

Slow and gentle exercises, such as walking and Tai chi, are suitable for PD patients (33, 34). Due to the disease itself and the side effects of drugs, patients experience decreased appetite and malnutrition. Providing patients with a reasonable diet and balanced nutrition is beneficial to them. The cost of PD medication is considerable and exceeds the average economic capacity for patients, especially considering the price of anti-parkinsonism medication and the costs of care (35). However, our results show that the financial burden is far less weighty than the psychological and work/society burdens of PD patients. We should also provide the necessary psychological counseling to patients who are still working. For advanced patients, a social platform on which they can communicate with and inspire each other is needed. With the rapid development of the Internet, patient support networks should be screened regularly, and developing a new digitalization process involving wearable devices, healthcare electronics and smartphone medical application services could contribute to enhancing quality of life (36).

Insights from this study are a valuable addition to existing knowledge about PD patients' experiences (comparisons between the SML and survey study are shown in Supplementary Table 1). However, there are some limitations. Everything new that is discovered in an SML study may need to be further probed in a survey or qualitative research study, ideally in a private online community, to achieve on-demand insights. The relationships between themes and keywords were preset. Some non-motor symptoms (e.g., a variety of behavioral problems including pathological gambling, compulsive shopping and eating, hypersexuality and other impulse-control disorders that have a relatively high frequency in PD) were not analyzed in the overall analysis but were analyzed only for specific drugs. Additionally, information on lifestyle factors, such as utilization of tobacco or alcohol or caffeine, was not analyzed because of the low number of mentions. Furthermore, the expressions of patients may be a function of how motivated they are to use social media, which can be affected by their income, education and network conditions. Negative perceptions may be vocalized more often than positive perceptions/experiences.

## Conclusion

This SML study provided evidence supporting the social integration of individuals living with PD. It is imperative to plan individualized therapies, education strategies and social support for the management of PD patients. The construction and maintenance of social media platforms for PD patients requires medical staff or even specialty staff, and this is also an effective approach to solving the unfair distribution of medical resources in chronic disease management.

## Data Availability Statement

The original contributions presented in the study are included in the article/Supplementary Material, further inquiries can be directed to the corresponding author.

## Ethics Statement

This study was reviewed by the Research Ethics Committee, Ruijin Hospital affiliated with Shanghai Jiaotong University School of Medicine, and it was deemed exempt from review as it did not meet the definition of “human or animal subject research.”

## Author Contributions

YQ and YZ: default key words, clinical analyses, and manuscript writing. XH, SX, XY, and CM: content summary and manual classification. XL and MQ: web search and statistical analyses. QX: study design, project management, financial support, and manuscript revision. All authors contributed to the article and approved the submitted version.

## Conflict of Interest

XL and MQ were employed by the company Huimei Digital Tech (Beijing) Co., Ltd. The remaining authors declare that the research was conducted in the absence of any commercial or financial relationships that could be construed as a potential conflict of interest.
