# Supplementary material for: Findings in Chinese Patients With Parkinson's Disease: A Content Analysis From the SML Study
Source: Front Psychiatry. 2021 Feb 2;12:615743. doi: 10.3389/fpsyt.2021.615743 (PMC7884465; doi:10.3389/fpsyt.2021.615743)
Supplement: Supplementary file 1 [file Data_Sheet_1.pdf]

Findings in Chinese patients with Parkinson's disease: a content analysis from the SML study

Supplementary materials

Yiwei Qian, M.D., Ph.D.[1,†], Yi Zhang, M.D.[1,†], Xiaoqin He, M.D.[1], Shaoqing Xu, M.D., Ph.D.[1], Xiaodong Yang, M.D., Ph.D.[1], Chengjun Mo, M.D.[1], Xiaomeng Lu, B.Eng.[2], Mengjuan Qiu, M.Eng.[2], Qin Xiao M.D., Ph.D.[1,*]

[1]Department of Neurology, Ruijin Hospital, Shanghai Jiao Tong University School of Medicine, Shanghai, 200025, P.R. China

[2]Department of Digital, Huimei Digital Tech (Beijing) Co., Ltd, Beijing, 200025, P.R. China

†These authors contributed equally to this work.

[*]Correspondence and requests for materials should be addressed to Qin Xiao (xq10537@rjh.com.cn).

Supplementary Table 1 Comparison between the social media listening study and the survey study

	social media listening study	survey study
Main source of the patients	On-line, no region limitation	Specific region with limited location
Research method	Content analysis	Observational study
Research content	Open content with related topic	Specific content with related topic
Sample size	Big	Limited
Information accuracy	Imprecise	Accurate
Purpose and Significance	Perspective, unsolved problems and unmet needs of the patients	Doctor’s concerns about the symptoms or medication of the disease

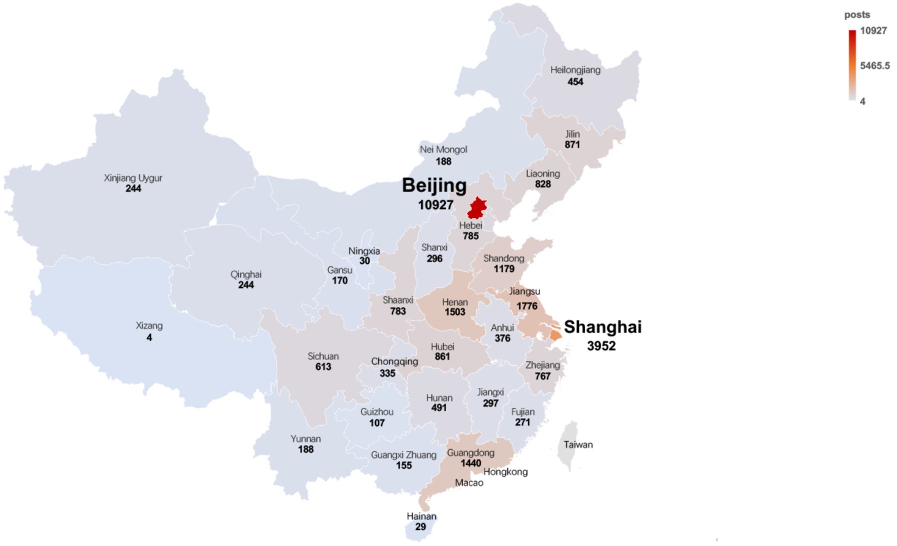

Supplementary Figure 1. The geographical distribution of the posts included in this study.

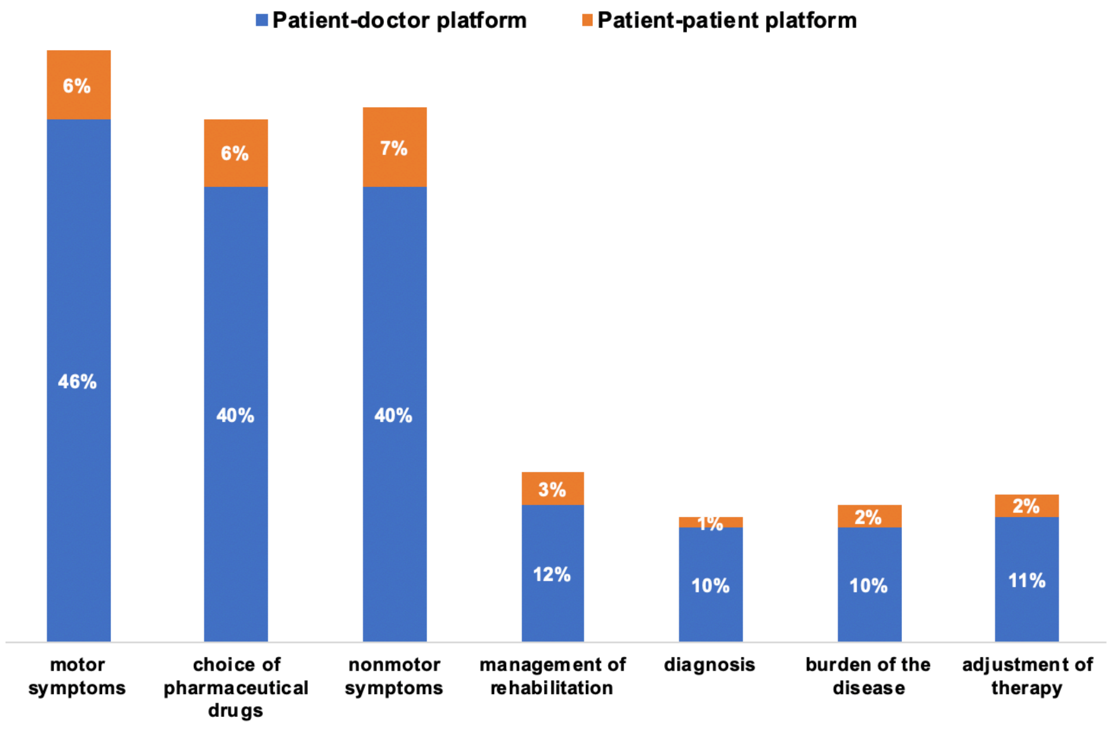

Supplementary Figure 2. The distributed differently extracted major topics in the two platforms.

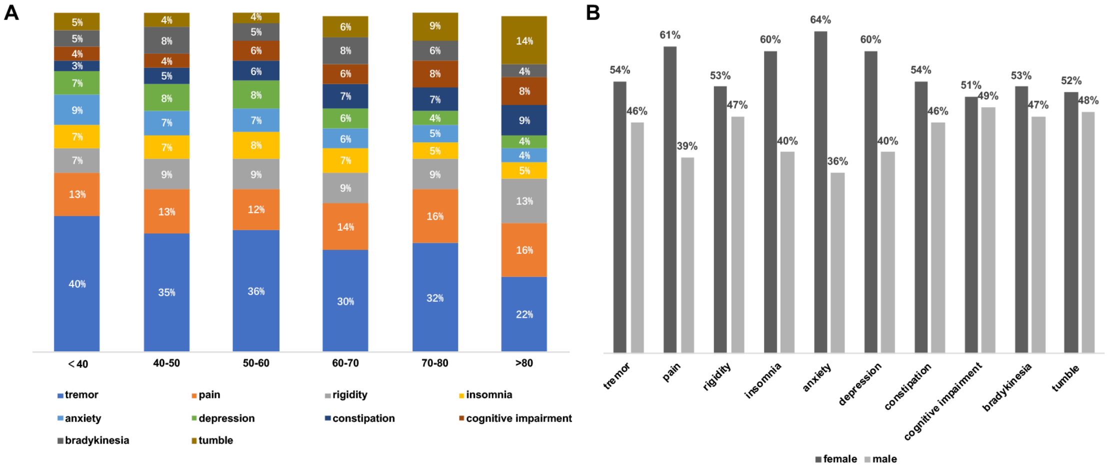

Supplementary Figure 3. The top 10 symptoms mentioned by patients in different age groups (A) and sex groups (B).

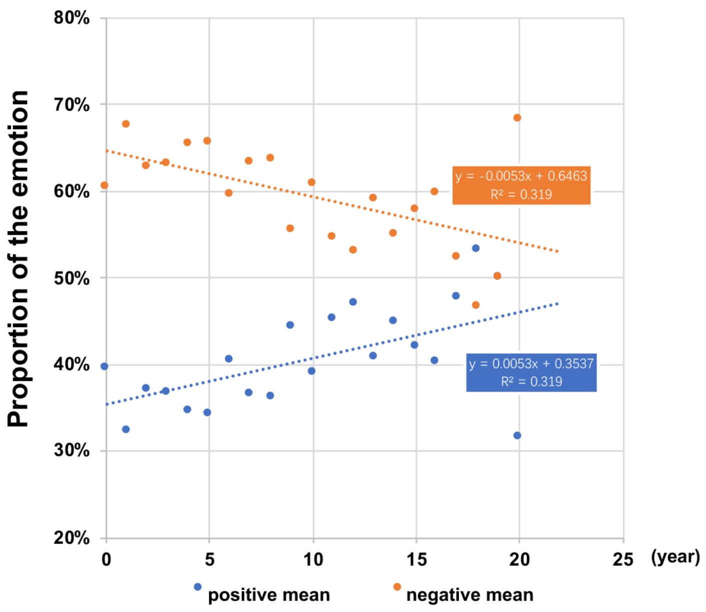

Supplementary Figure 4. Associations between positive and negative emotions and disease duration in PD patients. Results were detected by performing a naive Bayes classifier. The orange plot represents the mean values for negative emotions in each disease stage. The blue plot represents the mean values for positive emotions in each disease stage.

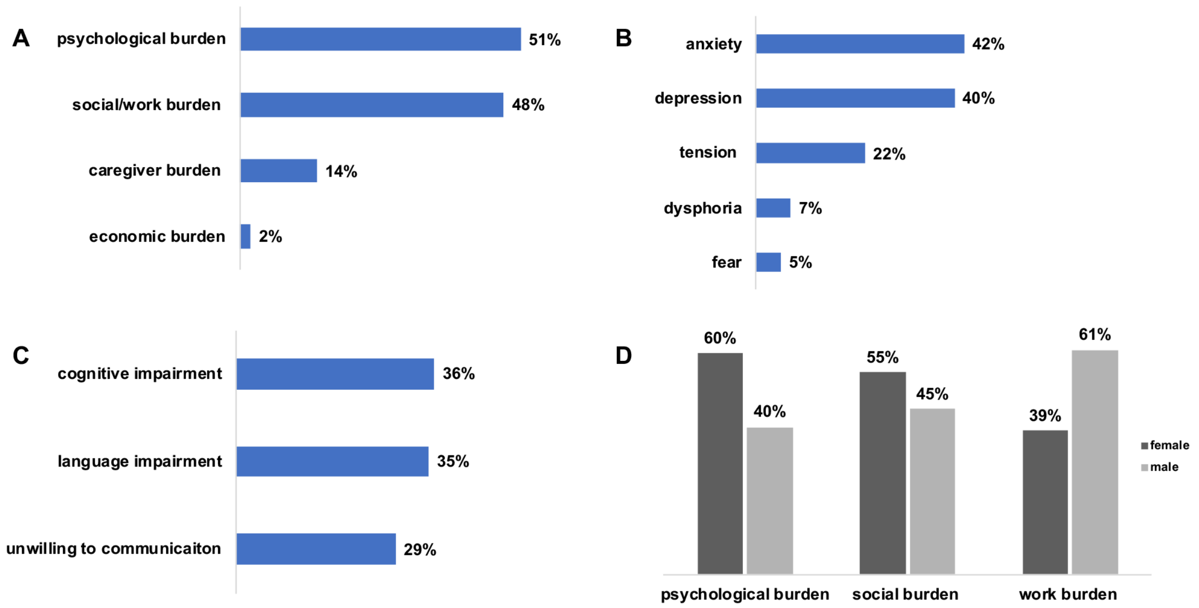

Supplementary Figure 5. Mentions in posts that pertained to burdens (A), details of mentions related to psychological burdens (B) and social burdens (C), burdens according to sex (D).

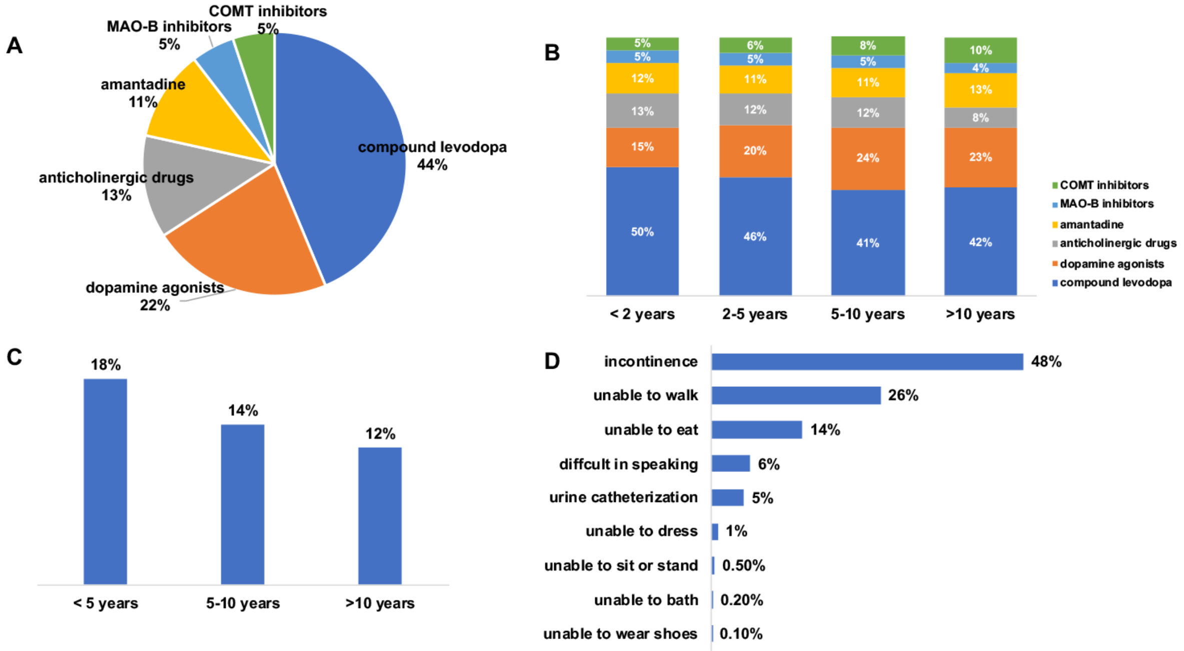

Supplementary Figure 6. Mentions in posts pertaining to management, including the main classifications of drug medication (A), the drug medications of patients with different disease durations (B), rehabilitation among patients with different disease durations (C), and in problems in daily activity abilities that impacted patients' quality of life (D).
